# Mr. Hill upon Caustic Issues in an Affection of the Spine

**Published:** 1813-03

**Authors:** G. N. Hill

**Affiliations:** Chester


*To the Editors of the Medical and Physical Journal.*

GENTLEMEN,

PERMIT me to recal to your attention the case of J. Edwards, recorded in No. 86 of your work. For the information of such of your readers as may not have perused the narrative in April 1806, or who have it not now in their power to refer to it, I beg permission to re-state the principal circumstances which led to the successful use of caustic issues on each side the lumbar vertebræ.

The remote causes which appeared to act in the production of a useless state of the lower limbs, with much defect in the upper, were violent and sudden exertions in his trade as a stone-mason; and the immediate or exciting ones, exposure to cold subsequent to intoxication, whilst riding near 100 miles on the top of a coach. The first fruits of the application of these causes became manifest at the age of forty-eight, by obscure pain at the lower part of the back, loss of appetite, constant feverishness, general weakness, and peculiar weariness of the legs, with inability to turn in bed; the sudden and total loss of the use of his right right leg and left arm. Both feet soon became stiff and pointed; the worst leg drawn towards the buttock with great stiffness of the knee, the other leg soon becoming similarly affected, he lay altogether helpless on his back.

I first saw him in Nov. 1802, after every thing had been done for him which the practice of two infirmaries could suggest, without any apparent beneficial result. After close attention for a year and a half with no better success, he returned to our infirmary, and I did not see him again till spring 1805. I now examined his back with great care, but could discover no curvature in any part of the spine. He had obscure pain from the commencement of the lumbar vertebræ downwards, which he said was trifling until he was terribly jolted in a cart going and returning twelve miles to his parish. He could retain his fœces and urine, but rendered the latter every half hour; was greatly constipated and very weak. I applied, in June 1805, a caustic on each side of the lumbar vertebræ, the disease having existed near four years: in six months he was able to walk, and in the following spring he returned to his work for a short time.

Believing himself well, he neglected his issues. They were dried up early in the ensuing summer, so that they were employed but about twelve months. Before the end of the winter his appetite diminished; from being fat and well-looking, he became thin and sallow, felt return of stiffness in his knees and ancles, weariness of his limbs, and general debility, which led him to the desire of lying constantly on the bed with his knees drawn up. I now saw him once more, and earnestly urged the replacement of the issues. He did not consent till the end of October 1807, when he was nearly as helpless as ever, with more pain in his back, and abdominal fullness—great costiveness, hectic fever, and dyspnœa tiresome—continual rendering of urine—the issues discharged little—he grew low-spirited, sunk fast, and lived but till March 1808.

No arguments could induce his relatives to let me open the body. Externally the abdomen appeared full; his back, from an apparent spinous projection of the last dorsal vertebræ downwards, was now more hollow than natural; the sacrum projecting as is seen in ricketty children, where this bone seems, from its morbid state, to be incapable of sustaining the superincumbent weight, and is at the same time driven backwards by the enlarged lumbar vertebræ, which of course gives a projecting appearance: hence, as he lay, the nates and dorsal bones formed the resting points, the inter-space an arch quite clear of the bed, the knees drawn drawn up as high as possible, and as stiff as though they had never had motion.

Are not the following inferences fairly deducible from Edward's case?—1. That for a great length of time every means that art could suggest was tried to relieve this subject without any beneficial effect, until the caustic issues were used as a last resource. 2d. That, in a short time after their application, evident relief was obtained, and in twelve months from their insertion the use of the lower limbs was restored. 3d. That, not continuing the active remedy employed sufficiently long to remove the disease, relapse ensued, and death was the consequence.

Our great predecessor, Mr. Pott, observes, that, "The majority of those who labor under this disease are infants or young children; adults are by no means exempt from it, but I have never seen it at an age beyond forty." Edit. of 1783, p. 435. "That the caries with curvature and useless limbs, is most frequently of the cervical or dorsal vertebræ; the caries *without curve*, of the lumbal." P. 473. "That the ligaments connecting the vertebræ which formed the curve, were altered from a natural state by being thickened and relaxed; the bodies of those bones were palpably spread and enlarged in their texture." P. 408. "That, previous to the useless state of the lower limbs, and the alteration of the figure of the back-bone, there is a pre-disposing cause of both, consisting in a distempered state of the ligaments and bones." P. 409. "That the restoration of the spine to its natural figure depends much on the early administration of the help proposed." P. 421. "That the patients whom I have attended in the early part of the distemper, of *whatever age*, have all got well." P. 423. "That the first evidential symptoms of disease in adults was a sense of weakness in the back-bone, accompanied with a heavy dull kind of pain, coldness and numbness of the limbs, general lassitude, and a small degree of exercise inducing fatigue. P. 438. "That the primary and sole cause of all the mischief is a distempered state of the parts composing, or in immediate connection with, the spine, tending to caries. From this proceed all the ills, whether general or local: apparent," as in the curvature from within outwards; "or concealed" (p. 445), as in the curvature from without inwards. "That this deformity, with affection of the limbs, although different in degree and extent in different people, yet was uniform in one circumstance, which was, that the curvature always was from within outwards, the smallest being attended with the same consequences as the largest." P. 451. "That the state of the cartilages is also subject to great variety." P. 452.

 "That

"That it is a distemper subject to great variety, from which variety its very different symptoms and events in different subjects can alone be accounted for." P. 474.

Five cases have occurred to my notice since that of Edwards, and all of them differing in the external appearance of the spine from these so accurately described by Mr. Pott, yet differing but little in other respects, and not in the least as to the propriety and consequences of the treatment recommended by that great man, or the fatality of neglect of its early application.

The importance of the subject will justify the propriety of reciting a short history of these, previously observing that singular ones will be far more apt to lead to neglect and ultimate destruction than those detailed by our great predecessor, because they are necessarily involved in more obscurity from first to last. 1st, The subjects of four of the number were above forty years of age; the fifth between thirty and forty, which circumstance constitutes the first observable distinction between this form of the disease, and that treated by Mr. P. The 2nd marked deviation is, that the osseous affection has been, in every case, situated in the lumbar, and not the dorsal or cervical, vertebræ, sometimes including the upper part of the sacrum. 3dly, That the patients laboring under the lumbar form of the disease, have the curvature from without inwards, a reverse situation to those treated by Mr. P. 4thly, That, until nearly the close of the disease, the spine does not manifest to the eye or the touch any, or scarce any, deviation from its healthy structure. 5thly, That the distressing tightness across the stomach is a rare symptom in the internal lumbar distortion, whilst in those of the dorsum and neck it is always present. 6thly, That, though the general leading symptoms accompanying the early stage of spinal local derangement where there is no external appearance, and that where osseous deformity is obvious, are similar, yet they are, until the near approach of death, always milder, more variable, and far less rapid in their course to a fatal termination. 7thly. The power of motion, when in bed, and of sitting in an easy chair inclining forwards, for a short time, remains to nearly the last with those who have internal distortion, though they cannot stand better than the opposite cases; but is generally early lost in the other forms of the disease.

A short history of each case will illustrate these conclusions, drawn from the whole evidence afforded. The next to that of Edwards was a female, who, when I first saw her, was so far advanced towards the last scene that the caustic issues were applied merely as a forlorn hope. They, however, however, produced such a degree of partial good effect upon the useless limbs, with the attendant symptoms, as to convince me nothing was wanting to have effected a cure but their earlier application. To the by-standers the case appeared in a different light: the re-action excited in the system was viewed by them as accelerating the sufferer's death. She had been nearly helpless for six years, and dated her first feelings of indisposition to have existed for above a year before that. This sufferer, like all the rest, would sit from half an hour to two hours in an easy chair, till within a short time of dissolution, but never without having a low table before her to recline the upper parts of the body upon. The sense of uneasiness, followed by inability to sit longer, always commenced about the end of the dorsal vertebræ. She could not retain her urine in the least degree, was constipated to an extreme, and when an evacuation was procured, unless the fœces came off very large and hard, their passing the anus was scarcely perceived. She died under similar circumstances to Edwards, the body presenting the same form after death as his did. Permission to open it could not be obtained.

The third subject was a male. The affection in the back and lower limbs had commenced very gradually twelve years before. I saw him at the age of twenty-eight, after suffering all the symptoms of lumbar abscess, followed by a considerable discharge of matter per anum. Soon after the cessation of this evacuation, those symptoms already enumerated gradually came forward, but so slowly that nature seemed to become accommodated to the change; and, when desired to attend him, I could discover no spinal deformity save a small degree of seeming elevation of the spinous process of the last dorsal vertebræ, and considerable concavity of the lumbar, with proportionate projection of the sacrum. He had little command of urine, obstinate constipation, with perpetual coldness of his lower limbs. He was a timid man, and though anxious to gain the use of his legs, he was afraid of the caustics: hence two very small ones were applied, but were never suffered to be renewed so as to induce much discharge. In a short time they were suffered to cicatrize, and he remains much the same.

The two next were one of each sex, neither of them of longer duration than two years, but both having the symptoms described in Edwards's case as decisive as possible, and both having been treated by as great a variety of medical means as the invention of various practitioners could devise, and which it would be tedious now to enumerate. When I first saw them they were in nearly as deplorable a situation as any of those so well described by Mr. Pott, and who recovered; yet in neither could any of those external evidences marking deviation from healthy arrangement, so minutely described by that great man, be discovered by the eye or the touch. After two years' existence in the one case, and a year and half in the other, the last dorsal vertebra was, in both, seen to form a small projection, with a proportionate depression of the first lumbar, and from this spot a general bending or concavity down to the sacrum, with full nates; yet every other general symptom was as unequivocally correct as to leave no doubts on my mind of the existence of osseous disease, as clearly as though the spinal curvature had been from within outwards. Very little was done for either after the formation of the caustic issues, regulating the bowels excepted, and both fully recovered.

The last case of this description, and the one to which I am desirous to excite particular attention, terminated fatally a few weeks since; and, as a partial inspection of the body after death was permitted, the history becomes the more interesting. The subject had been an athletic man, yet, what is called, of a nervous habit, forty-six years of age at his death (by trade a master stone-mason). Seven years previous to this event, he first began to complain of obscure uneasiness in the small of his back, which he attributed to two or three strains from repeatedly lifting heavy stones suddenly. This uneasiness did not amount to pain of a long time, but induced such an increased tremulus of the nerves; a weariness of the legs and thighs, with pain in them; general sinking, as to cause him to sit down often during the day, and go to bed earlier than usual. Rest removed these feelings, and he continued working occasionally for two years and half afterwards, when, being in Liverpool streets, he became rather suddenly worse, incapable of walking steadily, and prone to fall from the smallest impediment, and soon after to actual sinking and dropping to the ground, without any assignable external cause whatever, appearing to spectators as though inebriated, having lost the power of regulating the action of the muscles of the lower limbs. He still persisted in going out to direct his men, though less and less daily; until, at length, having one morning crawled about two hundred yards from his own house, he sunk without a stagger to the earth, and was re-conducted home never to walk more. This was about three years since. A great variety of general means were used for his recovery by an eminent physician and surgeon, comprehending perpetual blistering of the back, and electricity. His spine was carefully examined, without any curvature being discovered, or

 much much pain on pressure. He could retain both urine and fœces, but was obstinately costive, and never experienced an erection. After having taken to his bed for a few months, paralytic affection seemed to have seized his right side, including the arm. General emaciation gradually followed, with a most obstinate constipation and tormenting enuresis, the lower limbs becoming more torpid, cold, stiff, and useless; but the closest examination of the spine afforded no evidence that there was any vertebral derangement. Heavy pressure would excite simple uneasiness, but nothing more. His appetite was good; and, notwithstanding the complete immobility of the lumbar vertebræ, he could shuffle out of bed without assistance; and, when lifted into an easy chair, could sit with the upper part of the body resting on a table or chair before him for an hour or longer; but, upon asking him to continue, he would say his back was too weak and uneasy, and describe this feeling as situated in the lower part of the dorsal vertebræ. Thus he continued without any material variation till within a few weeks of his death, almost altogether in bed, only growing more helpless and difficult to be removed.

In April last, I was desired to see him. Upon looking at the back, the spinous process of the last of the dorsal vertebræ presented a small knob to view, which gave the first of the lumbar a sunken appearance; pressure here excited considerable uneasiness, and endeavoring now to sit up as long as usual, was sure to invite pain in this spot, and thence downwards, with general irritation, irresistibly compelling his return to bed. The lumbar vertebræ down to the sacrum, together with that bone, presented the appearance already described; the limbs corresponded with the well-known state so often mentioned; he was rendering his urine every quarter of an hour; and, if the feeble but constant inclination was not instantly obeyed, it came away involuntarily, as had long been the case, during sleep. A dark scybalous dry fœtid evacuation, once a-week, or in ten days, was the ordinary state of his bowels; he had occasional chills and hectic flushes, with a feeble pulse and a whitish tongue. I concluded to try the caustics as a forlorn hope, convinced that they afforded the only chance of rescuing the sufferer from the grave, guarding my opinion with a doubtful prognostic. They were applied the 30th of April; they began to discharge a little in six weeks, but the eschars did not separate till the middle of July. He complained of spasmodic twitchings in his limbs from early in June, with pains in the joints from the hips downwards.

Having been long aware of the importance of a healthy state of the intestines in every disease, I directed a small dose of the pil. hydrarg. at night occasionally, with a few grains of aloes and rhubarb the following day, by which means his evacuations became better in appearance and more frequent; but, when at all soft, he had no sensation of their passing the anus. By the time the issues began to discharge freely, these medicines had become unnecessary; he had a tendency to diarrhœa, and his urinary evacuations were less frequent, the limbs were not quite so benumbed. This was all the advance he ever made. The discharge from the issues had been copious and good for about a month, with the usual luxuriance of central fungus, and disposition to heal at the edges; but about that time they flattened, appeared glassy, discharged a thin offensive sordes, his appetite diminished, thirst increased, tongue became brown, furred, and foul; gums sordid, fœtid breath, and hectic fever; gradually worse, with violent sweats, occasionally cold; no additional pain, but that of the limbs somewhat increased. He had a very quick feeble pulse, with a hollow cough, laborious respiration, and restless nights; in short it was evident that the disease, having existed seven years, was, in the emphatic language of the sagacious Pott, become "an over-match for the remedy." A situation was advancing which is but too common, loading our minds with unavailing regret.*

At this period a paraphymosis was first perceived to exist; he made no complaints of it, though the stricture was considerable. A couple of leeches reduced the swelling, and a cold solution of Zinc. Sulph. so far removed it as to render any operation needless. His urine and stools came off insensibly; but, after going on thus for a fortnight, a tumor above the pubes was perceived, and he complained of pain in the part. On examination I found it was the bladder not lying central below the umbilicus, but nearly in the right groin; fomentation and camphor liniment relieved him; still, in a few hours, it was larger than ever, very little urine having flowed off in the last 16 hours. With some difficulty I introduced a bougie into the bladder, and soon afterwards a metal catheter; about a pint of urine followed, but I could not completely empty the bladder by turning the instrument in any direction. In 12 hours it was become far more full

* Do we not daily find that the very same curative means applied to a patient at one period of a disease, will excite a new action in the system, and prove restorative; whilst the very same means, used at another period, will accelerate a fatal event? In the one case the remedy is an "over-match" for the disease; in the other, the reverse.

than than before; I could not introduce the same catheter without using more force than was justifiable, the impediment was in the neck of the bladder. I took one of the elastic catheters recommended by Mr. Home, and, after having passed it to the obstructing spot, upon withdrawing the stilet it slipped in,* and upwards of three pints of urine came away. I left the instrument in the bladder for some days, and on taking it out the urine continued to flow involuntarily the remainder of life, but never so as to empty the bladder entirely, there was always a fulness in the right groin; his hectic fever increased, with an intolerable thirst, profuse sweats, hollow cough, and labored respiration to the last moment, which arrived the 23d of September.

Under a solemn injunction to do very little, I was permitted to examine the abdomen. As the body lay on the back, the lower part of the spine formed the same arched appearance as occurred in Edwards's case. With the kind assistance of Mr. Titley, one of the surgeons to our city infirmary, I opened the abdomen; after remarking that it had not the lank sunken appearance which is common to subjects who die greatly emaciated, but it had a gentle fulness from above the umbilicus to the pubes, we found a very small omentum lying altogether on the stomach; this organ, the bowels, liver, and the other viscera, presented the usual appearances found in exhausted bodies; the gall-bladder diminutive, the kidneys deprived of fat, and rather small urinary bladder, thick, and lying to the right groin; all the lumbar vertebræ greatly enlarged, a line drawn from side to side over the last measured nine and a half inches; the enlargement commenced very abruptly about the first lumbar bone, which caused the last dorsal vertebræ to have a sunken feel; it likewise felt denuded and rough. The space between the lowest of the lumbar bones and the pubes was very small; there was no unusual inflation of the intestines, very little

* I avail myself of this opportunity to say a word respecting Mr. Home's catheter with iron stilet. When I had introduced the instrument into the bladder, it was with great difficulty the stilet could be withdrawn, and, when effected, the curvature given to the catheter was so great as to shorten it in such a manner, that, having but a small metallic top with rings for the tapes, and this coming off, the instrument slipped down the urethra so low that it was with difficulty recovered. Now, by making the stilet a little less, and plating it with silver; rust, and liability to become fixed, would be avoided. The metallic ring at the upper extremity might be made larger, of a conical shape, and be rivetted on; indeed, was the catheter two inches longer, it would be a material advantage.

fluid fluid in the abdomen, the interarticular cartilages were very wide and nearly as firm as bone, whilst the bodies of the vertebræ felt rugous, and appeared less than natural, or as if from their former texture they had not so speedily partaken of the general enlargement. Our examination was limited to what had now been done, but it was sufficiently confirmative of the true nature of the disease, as described in the following extract from Mr. P.—" The generally-received opinion, therefore, that all the attending symptoms are derived from the external curvature considered abstractedly, is by no means founded in truth, and may be productive of very erroneous conduct." P. 475.

In the dissections made by Mr. P., where the curvature was the reverse of what I have now attempted to describe, though when neglected or overlooked a similar result is inevitable, he observes, " The bodies of the vertebræ concerned in the curve were larger than they should be, and than those above and below were, and their texture much more open and spongy, which difference appeared immediately, before the parts covering them were dissected off." P. 411–420.

In his subsequent observations this admirable surgeon found occasion to remark, from extended experience, that " the bodies of the vertebræ are always diseased, which disease does not so properly enlarge as erode." P. 452.

Upon this observation it may be permitted me to remark, that all his subjects were young; that in such inflammation and destruction proceed much quicker than in older ones, where the bones have acquired their ultimate firmness before the latent predisposition to evil was excited into action; that erosion or caries, it must never be forgotten, is always the consequence of disease, not disease itself; that it is the disease preceding the caries which causes all the mischief; caries is not indispensibly necessary to the useless state of the lower limbs, to hectic fever, and to death, but the disease may exist with consequent caries in the lumbar vertebræ, without external curvature, as is illustrated by Mr. P. p. 472. And, that it may happen from enormous internal enlargement with commencing caries, we have just seen. Of the consequences of inflammation, and simple thickening of the soft parts of the bones, it is presumed no surgeon of any experience and attention can be ignorant: enlargement from these causes, where predisposition exists, may be carried to an amazing extent, terminating only in the final destruction of the sufferer, by wearing him out with useless endeavors to remove the original cause of all the accumulated misery. Osseous swellings of this description may be for a long period after their commencement viewed viewed as nearly local diseases, but they cannot be considered entirely so, seeing no disease exists independent of predisposition. A fall or a blow, a bruise or a strain, may be the exciting cause, though long concealed, of unsuspected internal derangement; a shock is given to some particular part, and in proportion to the natural action of which, and a certain state of the vessels in the neighbourhood, will be the extent and symptoms of the complaint; hence, when the lumbar vertebræ is the spot we have enlargement from without inwards, when the dorsal or cervical is commonly the reverse, except where machinery is used, and then there is no doubt but, so long as predisposition to enlargement continues, such mechanical evils, whilst they please the eye by counteracting external deformity, produce internal and the most dreadful sequelæ, as psoas abscess, &c. &c.

From a number of cases affording a great variety of serious consequences, I am well assured much calamitous evil may date its origin from injury sustained in the small of the back. The lumbar vertebræ, from their great motion in all the actions of the body, and from their being less protected than the other spiral bones, are more exposed to danger from sudden exertion and over-action in all the business of life, and imprudent boastings of strength, to which mankind are liable. Whenever such a case presents itself early, where the yet apparent mischief is but small, I think some have been saved from protracted suffering and serious consequences by the early use of leeches, cupping, and blistering, a simple diet, much rest, and mild aperients. On the least approach of continued fever, with those feelings in the lower extremities which Mr. P. has so accurately described as the precursors of distorted spine, with all its fatal consequences, I am convinced, that, whatever may be the external appearances of the back bones, the caustic issues cannot too soon be formed; nor is the accommodating principle in the system to be forgotten. The medulla spinalis, and the brain itself, will bear great pressure and derangement, if it be *gradually* inflicted, without evidencing the morbid change by convulsion or paralysis, for a very long period, and yet the destruction of the patient be firmly advancing. Hence this state, in the formidable cases I have been relating, has been overlooked for years, because there existed little or no external evidences in support of the real nature of the internal insidious mischief, and the exciting cause of which had been wholly forgotten.

The case just described being my first dissection in this form of the disease, I assign the same reasons for thus early publishing publishing as the excellent Pott did. I may also, without presumption, adopt the words of that great man in another respect, viz. that this form of the disease has hitherto been regarded too superficially; that we have been too prone to be satisfied with observing its external appearance only, without inquiring into its real nature; that there may exist external spinal curvature, and much consequent mischief, without caries, is well known; that there can be internal curvature without caries to any considerable extent, yet life be destroyed, is no less true.

I rest all I have said upon what he advanced: "A morbid state of parts previous to deformity must be allowed; every complaint of the living, and every appearance in the dead, prove it beyond contradiction or doubt. These complaints increase with the deformity, and decrease by early means used for relief; but, when used too late, or, from the inveteracy of the disease, the issues are found to be unequal to the wished-for effect, the general complaints receive no amendment, but increase until the patient sinks under them." P. 479. It could not escape the vigilant attention of Mr. P. that the disease was liable to shew itself under a variety of symptoms during life, and appearances after death; but, as before observed, all his subjects were young, and there is no account of any case in his work analogous to those I have related. The variety now spoken of differs from that treated by him in the particulars mentioned at p. 3.

From all which has been now said, I think no impartial judge will hesitate in allowing, that, if the sufferer, whose internal state was explored by dissection, had applied, in the outset of his malady, for relief from our art; and if, when he began to lose the power of walking steadily, the caustic issues had been formed, he would have stood a fair chance of recovery. I do not, by any means, presume to say that this form of spinal affection was wholly unnoticed by Mr. P. but it was only so in a general way, as, where he says, "Sometimes each of the distempered states of these parts is accompanied by a greater or less degree of deformity, *without any apparent disease* of the bones composing it;" and "sometimes the same bones are found to be carious, without any crookedness or alteration of figure." P. 468. He illustrated this truth by a drawing, and it is remarkable that the destruction shewn, without external deformity, was of the lumbar vertebræ. He laid great stress on the altered figure of the spine, as is evident from his 3d Axiom, p. 470, "That, when these complaints are not attended with an alteration of the figure of the back-bone, neither the real seat nor true nature of such distemper are pointed out by the general symptoms, and, consequently, that they frequently are unknown, at least while the patient lives."

I think the whole six cases now related afford sufficient evidence of the existence of general symptoms decidedly authorising the early application of the caustics; and that, when, through oversight, inattention, mistaken diagnostic, or pre-conceived theory, this remedy is not employed until the disease has become an "over-match" for it, surgery has failed in effecting all it is capable of effecting. In cases of external curvature, such unfortunate practice rarely happens: I say rarely, because I know it still does occasionally. But, in the cases now attempted to be described, no deviation from healthy shape in the bones is found, till very late in the journey of the disease to a fatal termination, and then may be easily overlooked. The whole existing symptoms, though so clearly described by Mr. P. and being always present in a greater or less degree, are deemed, as they were in the master stone-mason's case, a mortal nervous disease of the spinal marrow, which nothing could even arrest, much less cure. If, then, the form of the disease with external curvature may be mistaken for lumbago, rheumatism, &c. as was the case with a milliner at thirty years of age, in this city, how readily may the more obscure form of the same malady be wholly unascertained?

Remarks on the excellence of Mr. Pott's work would lead to great prolixity, if indulged, but one more observation may be permitted. He says, "Some predisposing cause must be looked for, in which (in my opinion) consists the very essence of the disease." P. 399. Of the correctness of this statement there can be no manner of doubt: the experience of half a century has confirmed it. The young woman mentioned above was cured, by the caustics, of the spinal disease, and enjoyed good health thirteen years; she was then gradually seized with the symptoms of hip enlargement, so well described by Mr. Edward Ford, and this the caustics removed, though, in consequence of the late application of the appropriate remedy, she had more lameness after than would have remained had it been earlier applied. The cure of the back-bone remained steady. It cannot be considered irrelevant to observe, that the caustic issue placed behind the great trochanter, has apparently been the means of removing the long-continued lameness in the hip joint, following a blow or fall, which, from its ambiguous nature, is indefinable, yet sufficiently mischievous to fix a painful impediment to free motion for years, or a long life.

From the whole then that has now been advanced on this interesting interesting subject, may not the following conclusions be fairly drawn:

1. That a useless state of the lower limbs, with a greater or less degree of concomitant symptoms, may be connected with internal deformity of the lumbar vertebræ, independant of any or very little external spinal deranged appearance.

2. That this affection of the lumbar vertebræ is more commonly met with after than before the meridian of life is attained; and, from this circumstance, and that of expecting always to find more or less of *external* spinal curvature in cases of useless lower limbs, medical men have overlooked the source of this form of disease.

3. That, although in this form of the disease no external curvature of the spine be present, yet it is attended with all the symptoms enumerated by Mr. Pott as accompanying the more common external curvature, that of the stricture at the stomach excepted; but that they are all milder in degree, and longer in producing a fatal effect, though ultimately as certainly destructive, if pre-disposition continue, as where the cervical or dorsal vertebræ are the immediate seats of the local mischief.

4. That, in all cases of a useless state of the lower limbs, where the general symptoms, so accurately described by Mr. Pott, are found to exist, let the age of the subject be what it may; yet, notwithstanding that the particular external curvature be wanting, the use of the remedies he has recommended are indispensably necessary.

5. That the chance of recovery in the lumbar spinous affection is nearly in exact proportion to the early or late application of the proper remedies, allowing for the difference of age; the younger having more chance of ultimate restoration after long delay than the older.

6. That, however long the disease may have existed, if the digestive organ have not absolutely failed, if hectic fever be not severe and constant, respiration laborious, and debility great, nor any additional disease present, it is proper practice to afford the patient a chance by the application of caustic issues, and the use of mild aperients and tonics.

